# Apocalypse Now!: From Freud, Through Lacan, to Stiegler’s Psychoanalytic ‘Survival Project

**DOI:** 10.1007/s11196-020-09715-8

**Published:** 2020-05-12

**Authors:** Mark Featherstone

**Affiliations:** Sociology, Keele University, Keele, ST55BG Staffordshire UK

**Keywords:** Freud, Lacan, Stiegler, Drive, Death, Computation, Survival

## Abstract

The objective of this article is to explore the value of psychoanalysis in the early twenty-first century through reference to Freud, Lacan, and Stiegler’s work on computational madness. In the first section of the article I consider the original objectives of psychoanalysis through reference to what I call Freud’s ‘normalisation project’, before exploring the critique of this discourse concerned with the defence of oedipal law through a discussion of the post-modern ‘individualisation project’ set out by Deleuze and Guattari and others. Tracking the development of ‘the individualisation project’ in history, I consider its connections with the cybernetic theories of Wiener and Shannon in the psycho-cyber-utopianism of the 1990s, before moving on to consider the other side of the psychoanalytic-cybernetic interaction through a discussion of Jacques Lacan’s rereading of Freud’s *Beyond the Pleasure Principle* in the second section of the article. In reading Lacan’s seminar on Freudian drive in terms of the cybernetic repression of death, I set up the conclusion to the article which involves a discussion of Bernard Stiegler’s ‘survival project’ that relies on a recognition of the limit of death in order to produce human significance and oppose the madness of our contemporary computational reality.

## The Freudian System and Beyond

What is the purpose of psychoanalysis in the early twenty-first century? In the late 19th and early twentieth century Freud’s invention was focused on strengthening the ego in the name of supporting the social self and finding ways to manage the pathological symptoms emerging from its primitive other, which Freud thought was poorly suited to living under the repressive structures of ‘normal’ society. The question of Freudian psychoanalysis was, therefore, concerned with how to (a) ensure the subjection of the self to the laws of civilization and (b) manage the fall out of socialisation through the treatment of the consequent symptoms of psychological distress. Thus, we might conclude that Freudian psychoanalysis was from the very start a ‘normalisation project’ focused upon ensuring human survival and working through the impacts caused by the transformation of the pre-human primitive into the social human. But by the mid to late twentieth century Freud’s critics, including Marcuse [1], Foucault [2], and Deleuze and Guattari [3], had started to argue that Freudian civilization was about more than survival and instead represented the psycho-politics of the old, phallic patriarchal order that needed to be swept aside to enable the youngsters to take over. In this respect the legitimacy of Freudian law was thrown into doubt and challenged by a new regulative principle: the law of the desire of the self. In this way the new counter-cultural law became about self-realisation and individual becoming in the face of a highly conservative system that was now thought to be illegitimate. The law of the Oedipal father had now been supplanted by the law of his children in the process of becoming. By the middle of the twentieth century, the struggle between these two regulative principles became central to the politics of psychoanalysis. This was the conflict between the ancient law of the authoritarian father who founded society and the new law of his rebellious children who wanted to escape from his repressive system in order to become their own people. Freud’s ‘normalisation project’ was now under serious threat from the rebels’ ‘individualisation project’, where the only law was the law to become who you are.

Although Christopher Lasch’s [4] criticism of the culture of narcissism showed that the novel turn towards self-realisation was not without its problems, by the end of the 1960s it was clear that Freudian conservatism was out of step with the emerging politics of the individual set upon the realisation of desire. In the face of the dark pessimism of the Freudian model, where the basic objective of the social system was to support the survival of the self that would surely self-destruct left to its own devices, the new psycho-politics of individualism started out looking like a recipe for utopia in respect of their refusal of limits and opposition to processes of normalisation. In this situation the role of society was minimal because the individual was now sovereign and restriction was considered illegitimate. Nobody wanted to listen to Dad in the new world because the law was about becoming, rather than limitation for the sake of others. However, the new utopia of becoming was short-lived because its expression through the market subjected individual freedom to the economic law of industrial production, consumption, production ad nauseam and trapped the freed self inside the endlessly repetitive capitalist system that seemed to have no outside. This is, of course, exactly what Adorno and Horkheimer [5] explained in their critique of the problem of industrially produced pseudo-individualism in the mid-twentieth century. Beyond Adorno and Horkheimer’s critique, however, the ‘individualisation project’ found new life in the 1990s in the cyber-utopianism that emerged alongside the democratisation of the internet. In this period virtual space was understood as a kind of lawless electronic frontier where cybernauts could explore their identity beyond the limits of the material body. In much the same way that Deleuze and Guattari’s [3] schizophrenic broke free of the law of the father by taking flight through the rhizomatic connections of desire, the cybernaut found the possibility of freedom inside the virtual network where the centre is everywhere and the boundary nowhere. In this new world the body started to look like something we could live without. Indeed, it was possible to maintain this utopian idea of the internet until the dot com crash in 2000, which led Silicon Valley start-ups to begin to look for new ways to capitalise on the newly networked self. At this point the complete freedom of the virtual cybernetic self started to fold back towards the paradox of the techno-pseudo-individual who feels completely free, but is in actual fact a servo-mechanism in a vast system of surveillance, behaviour modification, and profiteering through the translation of human experience into valuable data [6]. Enter the new law of computational identity, and the situation of the freed self within a new behavioural utopia where algorithmic certainty is more important than human freedom, which I want to return to later in this piece through discussion of Stiegler’s [7] high-tech nightmare.

Before we think about Stiegler, however, let us consider the same high-tech nightmare in Shoshana Zuboff’s [6] work. Beyond Adorno and Horkheimer’s [5] critique of consumer capitalism, and on the other side of the cyber-utopianism of the early 1990s where the individual was completely free to be who they wanted to be in the new virtual universe, Zuboff explains the new law of computation and the commodification of data in her book on surveillance capitalism. In Zuboff’s critique of the totally networked system, the problem of computational identity revolves around the idea that the self is endlessly in the process of being uploaded to a high tech, global network where it is transformed into data that is valuable for modelling behaviour and creating certainty for advertisers who want to know that their marketing hits the consumer right between the eyes. In this respect the effect of the network is to simultaneously (a) open up channels of communication between the self and the online world and (b) enclose the possibility of the now cybernetic self inside a circuit of surveillance, behaviour modification, and profit organised to ensure the production of late capitalist certainty. Thus the idea of individual freedom that evolved from the post-Freudian champions of the self up to the cyber-utopians is now subjected to a new law of computational certainty. The self is now a calculation. Under these conditions every move we make is predictable. This is, in my view, the challenge psychoanalysis must address in the early twenty-first century. Beyond Freud’s ‘normalisation project’, on the other side of the rebels’ ‘individualisation project’ that eventually found novel form in the cyber-utopianism of the 1990s, I want to suggest that the problem of psychoanalysis in the early twenty-first century is to develop a ‘survival project’ to save the self from destruction in computational systems that have the potential to calculate it, and every other form of organic life, out of existence. In order to explain what this ‘survival project’ might look like I turn to Stiegler’s [7] apocalyptic thinking.

While the problem of Freudian psychoanalysis was the social control of primitive man and the symptoms resulting from his repression in civilization, and the objective of Freud’s post-structural critics became about escape from the miserable fate of Oedipus, my argument is that the central question for psychoanalysis in the early twenty-first century revolves around responding to the ‘normal pathological’ situation brought about by the uploading of the individual to a network that is no longer able to sustain meaningful life (and potentially life in itself) because it is entirely characterised by technological processes of surveillance, datafication, behaviour modification, and instrumentalisation [6]. Under these conditions the problem that confronts psychoanalysis is less the need for repression to control the primitive self (‘the normalisation project’) or escape from psychological control in the name of the imagination (‘the individualisation project’), but rather the paradox of hyper-individualism where the self collapses under an excess/lack of freedom which we might understand in terms of the replacement of meaningful social structures by technological systems that no longer speak to the human need for significance to make sense of the narratives that connect past, present, and possible liveable futures. Here, the problem of the hyper-, or, we might say more-than-individual concerns the fatal tension between (a) absolute freedom from constraint brought about by the universalisation of the ideology of consumption and the consequent collapse of social structure that had been set up around the law of the need for the limitation of the self and (b) complete determinism resulting from the emergence of technological and algorithmic forms of organisation that advance consumption by wiring the individual into circuits of surveillance and behaviour modification. Thus the individual is absolutely free to consume and pursue its deepest darkest, most perverse desires, but at the same time condemned to a life of meaninglessness by the withdrawal of social symbolic systems towards a high level of technological abstraction that no longer carries human significance. In this situation the individual is completely free, but at the same time wired into a cybernetic system where its behaviour is translated into code that renders its movements entirely predictable into an endless machinic future where nothing ever changes.

Cast out of the meaningful symbolic systems that have historically, to use Winnicott’s [8] language, ‘held’ the individual and made them human, the self has no way to orientate itself in time or understand the connections between past, present, and potential futures that structure the possibility of free will. The result of this retreat of meaning is that the individual’s experience of their own development through time starts to atrophy into a kind of permanent present defined by the higher level of technological abstraction, algorithmic logic, and instrumentarian circuits that sit behind the virtual networks which appear to enable absolute freedom, but in actual fact reduce the now cybernetic self to a less/more than human servo-mechanism determined by surveillance, datafication, and behavioural control [6]. This is the computational law of the contemporary global economy. Caught in the computational bind between (a) the appearance of absolute freedom and (b) the unconscious sense of complete determinism without (c) the mediation of a social symbolic system to locate the self in temporal structures, the individual collapses towards the addictogenic logic of drive which offers no escape from its terminal situation, but simply confirms its subjection to the fatal mechanisation of the networked system that has no objective beyond its own (endless) reproduction. In the face of this situation the objective of a new psychoanalytic ‘survival project’ would be to save the cybernetic self from its fatal mechanisation through opposition to the addictogenic system where the behavioural reflex of unthinking consumerism stands in for freedom of thought, free will, and motivated social action based upon the reconstruction of significance within symbolic systems that operate on a human scale. In the discussion that follows I seek to outline this project through reference to the work of Stiegler and in particular his recent discussion of disruption and madness [7], but before I reach this point I want to start by exploring the complicity of psychoanalysis, technology, and cybernetics. The reason this reconstruction is important is because recognition of the history of what Lydia Liu [9] calls ‘the Freudian robot’ will shed light on the challenge Stiegler explores in his work and that I consider key to understanding the political role of psychoanalysis in the early twenty-first century.

Although Liu centres her study upon the relationship between Wiener, Shannon, and Lacan, she might have projected her discussion back further to consider the origins of the Freudian machine. In Liu’s work it appears that the Freudian robot was Lacan’s invention, but there is a sense in which Freud’s psychoanalytic self was always already becoming a technological organism comprising a primitive throwback (the Id), a forward facing social self (the ego), and a sociological other (the super-ego) able to ensure that the primitive in man never takes over, but instead somehow manages to follow the (Oedipal) law. In this vision of human psychology the self looks like a kind of communication and control system leading to the possibility that the history of psychoanalysis might be understood in terms of the exploration of the ways in which primitive man is subjected to sociological law through (a) historical communication systems (psycho-sexual development and socialisation through the family) that enable (b) social control (the incest taboo and its extension into the law of father) to operate more or less successfully. Of course, there would have been no need for psychoanalysis in the first place if this machine worked perfectly, and the history of analysis has always revolved around working on malfunctions in cases where the technological self fails and communication and control no longer function. In these cases Freud is a psychic engineer working out problems of communication (the failure of Oedipus) and control (lack of integration/normalisation) and looking for ways to repair the defective psychological mechanisms in the name of the functionality of the cybernetic psycho-social machine. Now it has not escaped the notice of key historians of psychoanalysis, including Friedrich Kittler [10] and Eric Santner [11], that in many of the most famous cases of psychoanalysis the metaphor of the psychic machine is fully revealed and requires very little interpretation. Let us reflect on the most famous of these cases for a moment.

First, consider Freud’s [12] Schreber were the paranoid judge struggles with ‘writing down machines’ controlled by his torturing God and the entire universe operates like a vast technological system set upon his torment. The metaphor of the machine is similarly revealed in Victor Tausk’s [13] study of the diabolical ‘influencing machine’ that looks like a coffin and ends up representing the automation and eventual death of Tausk’s patient, Natalija A. Developing Freud and Tausk later on in the twentieth century, Bruno Bettelheim’s [14] famous case study of ‘Joey, the mechanical boy’ makes it clear that the idea of the machine takes over when human life itself becomes unbearable. In the case of Joey a lack of parental love leads the youngster to find comfort in cardboard boxes and tinfoil devices that represent his mechanical escape from feeling and pain. In each of these cases the machine emerges revealing problems in the communication and control systems of the self that would otherwise ensure the normal running of Freud’s technological human. In much the same way that Heidegger’s [15] machine only reveals itself in its dysfunction—so, for example, I only become aware of my computer keyboard when the ‘F’ key sticks and I am estranged from the device in the act of typing that no longer simply happens—the malfunction of the Freudian machine reveals its mechanisation in the form of what Jeffrey Sconce [16] calls ‘the technical delusion’, where madness takes the form of a fantasy about controlling machines (including in contemporary society television and the internet). Although Sconce recognises the paradox of the technical delusion today, which is, of course, that one no longer necessarily needs to be paranoid to imagine that one’s movements are being closely watched by some remote power, he does not take the next step that would involve understanding the delusion of mechanisation as a recognition of the basic technological nature of the self made through its connection to society.

The value of making this point in the context of writing the history of the technical delusion would be to say that what the history of mechanical madness really reveals is the ongoing history of the collapse of the Freudian technological self in the face of the organic that endlessly resists technology and that what the problem of reality testing and the undecideability of sanity/insanity in the hyperconnected world illustrates is the possibility that the madness of Schreber, Natalija A., and Joey might no longer be pathological, but rather the normal psychological reality of life in a situation scarred by technological estrangement. In other words, in the new hyperconnected world we are all like Schreber and our pathology is a perfectly normal response to the madness of our high-tech social system that seeks to reduce the organic to the level of the machine. There is, I think, a sense in which Freud [12] was alive to the possibility that this is what might happen in the future (that is that the machines might take over) in his discussion of the problem of reality testing and the question of whether Schreber was onto something in his memoirs. Indeed, this might lead the reader of Freud to consider Schreber a key moment in early psychoanalytic thinking where the problem of the failure of the cybernetic human–machine interface shifts from one focused on the importance of the integration of the primitive self (where the problem of Thanatos is on the side of the unconscious) to one concerned with the extremism of the control machine itself (where death comes from the abusive interventions of the sadistic father and is only later internalised into the funereal self), which would involve the leap from Freud’s early ‘normalisation project’ to his own critics later ‘individualisation project’ that he (Freud) could never fully recognise or take on board. This is, of course, precisely how Eric Santner [11] reads the Schreber case in his *My Own Private Germany*. In Santner’s exploration of Schreber’s secret history of modernity, little Daniel fails to pass through Oedipus and become normal because of Dad’s sadistic therapeutic gymnastics, causing him to take off into paranoid delusion in order to save some semblance of a self. In other words, little Schreber imagines a completely mechanised universe set upon his surveillance and behaviour modification on the basis of his Dad’s extreme communication and control system organised to produce robotic men who could cope with the new modernity (industrialisation, urbanisation, technological communication). This had, we must not forget, led the original sociologists Marx, Weber, and Durkheim to imagine society shot through with estrangement, disenchantment, and anomie and there is secret history of the cybernetic metaphor in sociology (taking in Parsons and others) to be written. But that is for a separate article. For now we must stick to Freud and Santner’s Schreber.

In Santner’s [11] sociological analysis of the Schreber case the problem of child-rearing, trauma, and paranoia was not confined to little Daniel Paul, but rather ruined a whole generation of German kids leaving them feeling crushed, hardened to pain, and marked by a complete lack of empathy for others. In the context of Germany’s late nineteenth century hyper-masculine culture that found human weakness intolerable, Santner shows how these kids grew up into incomplete men who easily made the switch from masochism and the desire for domination to sadism and the drive to destroy the pathetic self in the other who started to look like a monstrous inhuman creature. In this way it is possible to see that the problem of the cybernetic proto-Nazi we find in the writings of Klaus Theweleit [17, 18] and Andreas Huyssen [19] was less an issue of the Freudian id in need of civilization and more about the social communication and control system itself slipping into over-drive. Although we can read about drive in Freud’s [20] work, there is no concept of ‘over-drive’ in psychoanalysis, which one would have to imagine represents what we reach when we move beyond drive. What is beyond drive? According to Freud [20] writing in *Beyond the Pleasure Principle* this is, of course, death itself which would explain everything about the link between the works of metalised writers such as Ernst Junger [21] and Ernst Von Salomon [22] and the eventual emergence of the suicidal Nazi state that was always rushing towards its own dead end. In other words, what Junger, Von Salomon and the Nazis wanted was to become machines, because this was preferable to the weakness of the human that they wanted to eliminate. But even though Freud’s post-war critics [1, 2, 3] recognised the need to move on from the father’s ‘normalisation project’, and understood that this was in many respects implicated in the paranoia of the state that saw enemy others everywhere, the problem is that their own ‘individualisation project’ never really escaped the gravitational field of the cybernetic self that grew up on Allies’ side of the war. We can see this most clearly in Deleuze and Guattari’s [3] critical transformation of the idea of mechanisation into machinism and their attempt to move beyond the paranoia of the state through the madness of the schizophrenic. Here, the Anti-Oedipal project embraces the circuitry of the cybernetic self by leaning on Bateson’s [23] machinic thinking, making use of the metaphor of the rhizome that extends in every direction, and pushing the idea of the desiring machine which centrally, Deleuze and Guattari point out, never escapes the pull of the paranoid state.

Given Deleuze and Guattari’s [3] conclusion that the schizophrenic can never break with the paranoid system once and for all, but only ever manage to reveal its difference from normal function, it is clear that where they separate from Freud is in finding virtue in malfunction, rather than in stepping outside of his cybernetic vision of psychology completely. In other words, *Anti-Oedipus* never challenges the basic idea of the technological self, but simply emphasises the possibilities of the ‘individualisation project’ inherent within the inevitable malfunctions of the Freudian machine, which is precisely why Deleuze and Guattari’s work was taken up so enthusiastically by the Californian cyber-utopians in the 1990s who (a) evoked the spirit of the counter-culture to move beyond the cybernetics of the Cold Warriors (b) and made the connection between the schizo and new techno-frontiersman working on his/her computer [24]. At no point, however, in this history of the shift from Freud’s ‘normalisation project’ to the rebels’ ‘individualisation project’ was the problem of the extension of the cybernetic idea (the mechanisation of the human) ever really in question, which is the point I want to suggest we reach in Stiegler’s [7] critique of techno-scientific excess and the complete robotisation of the self. Thus, I think Stiegler seeks to move beyond (a) Deleuze and Guattari’s schizophrenic and (b) the cyber-utopian idea of the experimental cybernaut because he recognises that these two versions of the ‘individualisation project’ represent the disruptive cutting edge of Freud’s functionalist self that has been (fatally) updated under the sign of Wiener and Shannon’s Cold War vision of communication and control in the contemporary surveillance capitalist system, which we might think about in terms of an extreme or hyper- ‘normalisation project’. Why ‘hyper’ or ‘more than normal’? The answer to this question relates to the extremism of the technology connect between human and machine. That is to say that what we find in Zuboff’s [6] critique of surveillance capitalism is a system that no longer normalises through Freudian cultural values, rules, and regulations that humans can understand, but rather an abstract machine that engineers absolute normality through the transformation of experience into data, algorithmic power, and behaviour modification.

In this respect, the new cybernetic system produces ‘more than’ normality, but Stiegler’s [7] point, which Zuboff never makes, is that the very extremism of this system means that what it ends up normalising is pathology itself, since it is impossible for the human to tolerate being transformed into a servo-mechanism without descending into pre-/post-human madness, precisely because the essence of humanity resides in the ability to exercise free will on the basis of an understanding of environmental conditions (the present) and the likely outcomes of motivated action (what happened in the past and how action might play out through the present in order to produce a future). Thus the new regime of ‘hyper-normalisation’ creates a situation that is also ‘less than’, ‘lacking’ or pathological in respect to normality, which is why Stiegler [7] connects this system to a kind of everyday madness and suggests that what is necessary to respond to this situation is less individualism (we are all pseudo-individuals today, caught somewhere between more/less than normality) and more a ‘survival project’, which could reconstruct the human from its organic basis founded upon its existence on earth that it then translates into a meaningful world. But how would this work? What would this ‘survival project’ look like and how could we (humans) save this utopian possibility from the current nihilistic system that seems endless? Before we reach Stiegler [7] and flesh out his theory of the high-tech apocalypse, it is important that we understand how psychoanalysis became fully robotised through consideration of Lacan’s [25] seminar on Freud’s theory of the compulsion to repeat and the fatalism of the letter that always reaches its destination.

## Lacan’s Cybernetic Turn

I think that it is possible to find the classic statement of the Freudian ‘normalisation project’ in *Civilization and its Discontents* [26] because this is where Freud most clearly explains the necessity of the social control of primitive man. In this essay Freud’s basic thesis is that humanity is too destructive to be left to its own devices and that some kind of society is essential to keep humans alive. In this respect, society is a kind of cybernetic life support machine set up to prevent humanity short-circuiting straight to self-destruct. However, the issue with this set up is that social security causes its own problems and Freud notes that rules, regulations, and prohibitions lead to enormous frustration. The basic emotion of civilized humanity is misery. We always want more, something else, and constantly seek it out. By virtue of our invention and ingenuity, Freud says that we have transformed ourselves into ‘Prosthetic Gods’, but this has made no difference to our levels of satisfaction. Humanity remains unhappy with its lot. But what is it that we really desire? In *Civilization and its Discontents* [26] Freud refers to the ‘oceanic feeling’ which describes the experience of the dissolution of the self and a profound sense of unity with existence that he understands in terms of death. Essentially, we want to escape ourselves (our separation) for some kind of peace. At this point, Freud clearly leans on his own speculations from a decade before when, in *Beyond the Pleasure Principle* [20]*,* he first raised the problem of humanity’s suicidal nature. In this essay Freud takes off from the observation of a child’s game, ‘fort/da’, which he suggests reveals the way humanity seeks to master the traumatic experience of lack through symbolism. ‘Fort/Da’ is a symbolic representation of the trauma of mother leaving /returning. What the kid can’t control in reality, he masters through play, thus solving the problem of trauma on a symbolic level. Unfortunately, Freud notes that since symbolism never really hits the spot, because it is impossible to resolve a traumatic situation that has already happened, the symbolic fix must be repeated endlessly in order to constantly defer traumatic recall. In Freud’s view, this is the compulsion to repeat, which resides on the other side of the pleasure principle. That is to say that if humans desire pleasure because pleasure makes them feel satisfied, then the compulsion to repeat looms into view because the search for pleasure is endlessly thwarted by the essential relationship between the human and trauma. We are our trauma and we cannot take this away. The role of Freudian psychoanalysis is to lead humans towards acceptance of this psychological fact.

Expanding upon this interpretation, Freud’s next move is to say that the compulsion to repeat is not simply a psychological condition concerned with the attempt to repress trauma, but that it is also reflective of an existential state he calls the inertia of being. What is the inertia of being? It turns out that the inertia of being describes the cyclical nature of existence itself, where organisms are born, live, and die in order that others might take their place, which Freud thinks is hard-wired into human psychology. The self is, after all, part of existence. Now the problem with the civilized human who reflects upon their lot, wonders about the meaning of existence, and searches for solutions to the experience of lack is that, in Freud’s view, they will soon become possessed by Thanatos or what he calls the death drive, unless some form of regulation is in place to prevent this happening. What this means is that when we take the risk to look behind the endless line of symbols we use to defer the experience of trauma what we find is that what we really want is to escape the self, which is born in traumatic separation. Behind the various symbols we use to hide from ourselves, we discover our constitutive trauma. This is what we want to escape. In other words, we enter drive when we look beyond the symbolic façade and confront the reality of what it would take to resolve our traumatic core. This is why Freud [20] writes about the condition of possession by Thanatos in terms of the Nirvana complex and evokes the Buddhist notion of the annihilation of the self to explain its function, with the important difference that he wants to contain the drive towards death in the name of saving the ego. Unlike the Buddhist who prefers the end of the self to the suffering of desire (the Buddhist concept of samsara), Freud thinks that misery is preferable to Thanatos and an orgy of destruction. Thus, humanity must be subject to cybernetic support and limitation in order to survive. Humanity cannot survive on its own. It needs its machines.

Now it is precisely this point about the fundamental machinism of humanity that Lacan [25] picks up on in his 1954–155 seminar on *Beyond the Pleasure Principle and the emergence of the ego*. According to Lacan, Freud’s greatest achievement was to reveal the decentred nature of humanity and show that we are never really ourselves. As Lacan says, the ‘I’ is always another. Reading Freud’s essay, Lacan starts by explaining that the desire for pleasure represents humanity’s orientation towards ‘the good’. While the search for pleasure represents progress (we’re endlessly moving forward), Lacan points out that the compulsion to repeat is regressive. In his view the compulsion to repeat is endless behaviour, in the sense that it is reactive and unthinking. We’re under the influence of something else here, something other than conscious thought, because the compulsion to repeat has no objective beyond its own reproduction. The compulsion to repeat is nothing but pure mechanisation. Recalling Heidegger’s [27] theory of the difference between humans, animals, and rocks, Lacan explains that if the animal is a machine unable to think its way out of its environment, the human is only slightly better off. This is because we are similarly ‘jammed’ by instinct, though we can at least escape our animal selves into the ego, which is the first object. This is essentially why we are excentric (external or outside) to ourselves. Unlike animals that are self-identical, Lacan points out that the human is separate from itself and only comes together in its mirror image. Thus recognition of the mirror image is the fundamental machine because it creates a feedback loop between the registers of the imaginary (the image of the body) and the real (the flesh of the organism) to enable the emergence of the self. For Lacan, this basic machine represents Freud’s [20] constancy principle (pleasure), since it provides some sense of a stable self (this is who I am!), which is then endlessly challenged by the repetitive search for the object lost in the trauma of separation. Since this lost object is itself lost to processes of repression which mean that we cannot remember what we really want, Lacan evokes the idea of the symbol and the symbolic order, which is his second machine. What this means is that the symbol stands in for the lost object. However, we know that the connection between the lost object and symbol could never be the result of a conscious decision, simply because the lost object is lost to repression, meaning that the process of symbolic construction must take place somewhere else, which is why Lacan’s second machine is the unconscious.

However, Lacan’s symbolic system is not simply unconscious in the sense that it is hidden somewhere in the subject’s mind. It is, instead, external to the self completely. In other words, it is no longer somewhere inside the head of the subject, but rather located in the communication and control system of language that sits outside of the self, leading Lacan to the techno-scientific conclusion that psychoanalysis is not a humanistic form of knowledge. This is, in Lacan’s view, why Freud moves into biology, rather than further into philosophy to explain the workings of the self. Beyond Hegel [28], who was never able to move beyond the anthropological ‘just so story’ of the master/slave dialectic, Lacan explains that Freud introduced the principle of energy into understandings of human psychology. Essentially, Freud turned the human into a machine in search of homeostatic equilibrium. Why is homeostasis impossible to achieve? The reason that we never reach equilibrium is because the dream machine continues to provide symbolic information about what we need to reach a state of systemic balance fuelling the mechanical compulsion to repeat. This is, essentially, Lacan’s [25] cybernetic re-reading of Freud, which he cements by explaining his idea of the symbolic law through reference to the Bell Telephone Company’s translation of communication into code. Noting that Bell needed to economise, and reduce the amount of information they had to pass through their phone lines, Lacan explains that the company sought to simplify communication. The impact of this move was to transform complex communication into simple code that was no longer transparent on its own terms. At this point he says that language became material, thing-like, objective and in need of interpretation/decoding to make sense. The point here is, of course, that psychoanalysis relies on a similar coding/decoding operation in the sense that the symbolic representations of the unconscious require reading by the analyst in order to expose their connection back to the lost object where meaning resides. The wider significance of the link between the psychoanalytic technique and cybernetic understandings of communication is, however, that beyond this or that representation of the lost object, the basic code of the symbolic order functions on the basis of whether or not the subject (a) possesses the object in symbolic form, before realising that (b) this, that, or the other version of the object is not the object itself, leading to further efforts to obtain the lost object leading back to a situation where (a) the subject believes they have finally found the lost object which similarly turns out to be (b) a symbolic representation of the thing, rather than the thing itself and so on ad infinitum. This is the cybernetic feedback loop of Freudian drive [20] operative within the Lacanian unconscious [25].

But beyond reflecting the circularity of Freudian drive, Lacan’s vision of the cybernetic nature of the unconscious also show how the infinite complexity of human language reduces towards a very simple binary code—satisfaction/dissatisfaction, being/not being, or plus/minus—leading him to suggest that the subject is a cybernetic machine endlessly switching between states of having/not having or not having/having, since the self is born in a state of deficit/lack/default (traumatic separation). Following this discussion, Lacan famously explains his cybernetic take on psychoanalysis through Edgar Allan Poe’s [29] short story, *The Purloined Letter*, where the lost object becomes the letter circulating between the Queen who loses the letter in the first place, Minister D who steals the letter and then proceeds to hide it in plain sight, and finally Dupin, who finally retrieves the letter and (we must assume) returns it to the Queen in order to complete the circle and ensure that the letter finds its way home. The key point of Lacan’s reading is that regardless of the movements of the letter, and who is in/out of possession of this prized (lost) object, it ends up finding its way back to its original owner. Despite the contingency involved in the play of dispossession/possession, which Lacan relates to the game of ‘odds and evens’, the unconscious rules of the game mean that things always end the same way. In ‘odds and evens’ the cast iron law of probability ensures that an infinite number of plays will always lead straight back to the start. Everything cycles back to the beginning in the end.

Given this conclusion, Poe’s [30] obsession with the end, and the funereal subject of Freud’s essay [20], it is surprising that Lacan never completely reveals or spells out the Freudian thanatology behind *The Purloined Letter*, which is that regardless of the route we take through life, we can never escape the ‘ghastly grim and ancient raven’ that cries out ‘nevermore’ [31]. In fact, Lacan tends to confirm this reading himself later in the seminar when he explains that what really lay behind the symbol, and the idea of the lost object, is lack and the basic anxiety that we feel before the endlessness of death. At this point Lacan [25] tells us that this is what the symbol ultimately hides: the Heideggerian lack inherent in being itself. However, Lacan fails to mention that this is precisely the insight that possessed Poe, even though he returns to his work later in his discussion when he mentions *The Facts in the Case of M. Valdemar* [32]—where the protagonist Valdemar meets his maker under hypnosis, but somehow retains the ability to live on to confirm his own death (‘I am dead’)—in order to restate the point that the symbol (the word) represents the minimal difference between life and death. What this means is that there is nothing, there is no error, there is only endless darkness, until the symbol emerges and invents the universe. This was, of course, Leibniz’s insight in the late seventeenth century when he wrote ‘omnibus ex nihilo ducendis sifficit uncum’ (to draw everything from nothing, one is enough) and essentially separated being from nothingness in binary code, where 0 is nothingness, and 1 is the creation of being [33]. Despite the creation of being, however, what Lacan’s [25] reference to Poe illustrates is that in the end there is no escape from the end. Regardless of its complexity, the symbol is ultimately a tombstone. Although the symbol may well represent the tomb, I think the issue of the extent of complexity is a very important one that needs unpacking because it has a bearing on the way Lacan understand the human relation to the machine. Towards the end of the seminar Lacan notes that the first symbol emerges from the body, meaning that it clearly refers back to corporeality and embodied experience, before increasing abstraction leads to the severing of the symbol-body connection. In the end the symbol has no body. At this point it is completely out on its own and the body is mute. It is interesting that Lacan passes over this point without elaborating on the difference this makes to humanity, which becomes ever more estranged from its symbolic inventions, because the most important impact of the increasing abstraction and simplification of communication is that it strips back what may very well be the necessary humanistic illusion that we are more than fleshy machines endlessly switching between being and not being and plunges cybernetic man straight into drive where elaborate civilizational codes start to break down before the mechanics of repetition and automation. This is, importantly, Stiegler’s [7] key point that I want to consider below. In his view we are not simply fleshy machines, but rather creatures that co-produce with technology. From this point of view, then, the key problem of the Lacanian idea of the subject is that it is too simplistic in respect that this figure is endlessly caught in a mechanical borderline state where it is either ‘on’ or ‘off’—similar to Claude Shannon famous suicide machine which has no other purpose than to relentlessly oppose its user by turning itself off [9]—and it cannot escape from the vicious circle of the eternal (robotic) return.

The problem of the Lacanian [24] vision of the self is, therefore, that from a humanistic perspective it is entirely robotised and erases the possibility that we might change our minds and break out of the nightmare of endless repetition. Despite this conclusion, however, there is a sense in which this conceptualisation of the self reflects the state of the individual in the early twenty-first century, particularly where a writer like Zuboff [6] who reflects on the computational violence of the hyperconnected society is concerned. Keeping in mind Shannon’s central place in the pre-history of the contemporary high-tech society, we might, therefore, support the conclusion of Martin Burckhardt and Dirk Hofer [33] who make the link between life in the new cybernetic society and BPD (borderline personality disorder), where one is constantly switching between being either completely null and void or hyperconnected to everybody and everything, and suggest that living on the borderline has become more or less normal in the dystopian online world caught between the Boolean alternatives of ‘all and nothing’. Similarly in the experience of bipolar disorder the mixed states of depressive-mania and manic-depression circulate endlessly meaning that the bipolar individual is never able to settle, focus, or think about life in terms of a narrative moving from the past through the present into the future. In other words, the bipolar person lives in a kind of vortex, a swirling permanent present torn between skyscraping highs and bottomless lows, and they cannot make sense of the world through long term projective thinking. Considering Poe’s own familiarity with the maelstrom of manic-depression [34, 35], it is, therefore, probably not very surprising that Lacan [25] finds himself referring to Poe to illustrate the psychology of his cybernetic self endlessly switching between states of being and not being, but what is perhaps more surprising is that he should end up normalising this vision of the self, particularly since Freud [20] clearly considered drive a pathological state and was deeply concerned about its destructive potential. What, then, led Lacan [25] to conclude that drive was normal? What led him to reduce Freud’s original psychoanalytic social control system to a post-human cybernetic switching machine? On the basis of reading Lydia Liu’s [9] book on the emergence of the Freudian robot we can support the claim that what led Lacan to break with Freud over the normality of drive was the influence of the original cybernetic theorists, and in particular Wiener and Shannon, who popularised the idea that the human mind could be thought about in terms of an elaborate computer program and saw no problem with the emergence of what we might call Boolean man. Although the pathological consequences of reading Freud through Wiener and Shannon to reach the conclusion that drive was somehow normal should have emerged from his reading of *Beyond the Pleasure Principle* [20], it is strange that Lacan barely mentions Thanatos or the ‘death’ drive in the seminar, even managing to more or less evade the subject of death in his reading of Poe, who was obsessed by the end! But why does Lacan’s repression of death in the context of this particular seminar matter?

The reason Lacan’s [25] conceptualisation of ‘drive without death’ is significant is because, I think, it opens up the possibility of thinking about a broad cultural shift over the course of the late twentieth/early twenty-first century towards a vision of ‘the dehumanisation of death’ in understandings of cybernetic humanity. This is what, I want to suggest, Stiegler [7] reveals in his work. In effect, then, I want to suggest that it may be possible to read Lacan’s normalisation of Freudian drive as symptomatic of a wider cultural move towards the normalisation of cybernetic violence in western culture that involves the reduction of the human to the level of a reactive servo-mechanism that collapses towards entropy, but never really thinks about its own definitive end. Although there was also a cyber-utopian thread running through the history of the twentieth century, which Fred Turner [36] uncovers in his work on the connection between the counterculture and cyberculture, my claim is that Lacan’s break with Freud in the name of marrying psychoanalysis with the computational thinking of Wiener and Shannon might be seen to represent a key moment in the history of thinking about human psychology in a high-tech society that explains the normalisation of the kind of mathematical violence Zuboff [6] writes about in her monumental work on surveillance capitalism.

Under these conditions, the law is no longer the Freudian law of *Civilization and its Discontents* [26] (the normalisation project) or the Deleuzo-Guattarian law of the schizophrenic of *Anti-Oedipus* [3] (the individualisation project), but rather the pre-/post-human law of Lacanian cybernetics that ends up (hyper-)normalising pathology (the cybernetic project). Regarding the emergence of this new law of wires, circuits, switches and electric flows, it is worth pointing out that there are several moments in Zuboff’s [6] book when she considers the problem of the normalisation of the commodification of human experience. She wonders—how is it that have we fallen into a cybernetic dystopia and come to think that this way of living is somehow normal? I want to suggest some kind of an answer to this question through my reading of Lacan’s [25] reading of Freud [20]. That is to say that the basic point of my reading of Lacan’s seminar on *Beyond the Pleasure Principle* is to suggest that it is possible to locate a key moment in the normalisation of the contemporary cybernetic self, in psychoanalytic thought at least, in Lacan’s normalisation of Freudian drive which we might then connect to the problem of hyper-normalisation (or the normalisation of madness) Stiegler [7] critiques in his work. Let me now take up Stiegler’s critique of high-tech society and his ‘survival project’, where he sets out his response to the new cybernetic dystopia that threatens to robotise humanity (and everything else organic) out of existence.

## Stiegler’s Apocalyptic ‘Survival Project’

In volume I of *Technics and Time* [37] Stiegler sets out his own cybernetic theory of humanity through reference to Plato’s retelling of the myth of Epimetheus in his *Protagoras* [38]. Against Marx who makes everything about Prometheus, Stiegler focuses on the role played by the other brother. Plato’s story goes:

Once upon a time there were just the gods; mortal beings did not yet exist. And when the appointed time came for them to come into being too, the gods moulded them within the earth, mixing together earth and fire and their compounds. And when they were about to bring them out into the light of day, they appointed Prometheus and Epimetheus to equip each kind with the powers it required. Epimetheus asked Prometheus to let him assign the powers himself. “Once I have assigned them,” he said, “you can inspect them”; so Prometheus agreed, and Epimetheus assigned the powers. To some creatures he gave strength, but not speed, while he equipped the weaker with speed. He gave some claws or horns, and for those without them he devised some other power for their preservation… Now Epimetheus, not being altogether wise, didn’t notice that he had used up all powers on the non-rational creatures; so last of all he was left with human kind, quite unprovided for, and he was at a loss what to do. As he was racking his brains Prometheus came to inspect the distribution, and saw the other creatures well provided for in every way, while man was naked and unshod, without any covering for his bed or any fangs or claws; and already the appointed day was at hand, on which man too had to come out of the earth to the light of day. Prometheus was at his wit’s end to find a means of preservation for mankind, so he stole from Hephaestus and Athena their technical skills along with the use of fire…and that was what he gave to man…And as a result man was well provided with resources for his life, but afterwards, so it is said, thanks to Epimetheus, Prometheus paid the penalty for theft [38, pp. 17–18].

According to Stiegler’s reading of Plato’s myth, humanity was always/already cybernetic and would never have survived without the machines Prometheus enabled them to create. In this theory humanity is born in default and cannot compete with animals with claws and teeth because of Epimetheus’ mistake which left humanity poorly equipped for life in the state of nature. However, Prometheus saves his brother’s bacon by stealing from the Gods in order to give humans a fighting chance. Thus commences the history of human civilization where problem-solving relies on natural ingenuity and the ability to make machines. Centrally Stiegler’s [37] thesis is that we have never reached the point where we have no more need for invention—the point of equilibrium, where no more development is necessary—because nature continues to thwart the possibility of technological utopia meaning that the need for innovation never ends. Humanity is, thus, endlessly in default. We make machines, that make problems, that require technological fixes and so on endlessly into the future. Although this was a sustainable situation for much of human history, Stiegler explains that modernity might be understood in terms of a kind of tipping point where technology stops becoming about the salvation of the human and starts to destroy its master. When this happens the logic of deferral fails and humanity is thrown into a permanent state of default (without switching), which Stiegler [39] looks to capture through the concept of ‘disorientation’, meaning that the human no longer feels at home, comfortable, or able to make sense of the technological world that seems to have left them behind. At this point Stiegler [37, 39] takes up Heidegger’s [40] famous critique of technology, enframing, and the forgetting of being to explain the estrangement of humanity from its own technology, but I think the shift he explains might equally describe the move that takes place in Lacan’s re-reading of Freudian drive in respect of the way that pre-/post-human mechanisation suddenly becomes normal leaving humanity stranded in a universe of wires, switches, circuits and electric currents. Referring back to Heidegger’s [40] distinction between ancient techne and modern technology, Stiegler [39] explains that modern technology suspends the history of necessary co-production, which saw humanity and machine become together in sympathy with the earth in order to survive, in favour of a new state of estrangement where machines develop and humans lose all sense of where these technological things came from or how they function. Under these conditions, the machine encourages men to start to look upon people and animals like so many things to be used and abused in the name of further development. We are now on road to the extinction of organic life.

In developing his thesis Stiegler [37, 39] refers to (a) Heidegger’s [40] critique of the replacement of the carpenter who uses tools to work with the grain of the wood by technology and the machine man who smashes nature into shape and (b) Marx’s [41] theory of the deskilling of craftspeople throughout the history of industrial capitalism to describe the experience of what he calls proletarianisation. In Stiegler’s [39] work the experience of proletarianisation, or what we might call ‘becoming stupid’, occurs in inverse proportion to the development of technology, leading to the conclusion that the emergence of cybernetic civilization involves profound psychological regression on the part of humanity. Mirroring Heidegger’s [42] critique of Nietzsche, where the utopianism of the ubermensch folds into the nihilism of the technological will to will and nothing more, or indeed Marx’s [43] theory of industrial capitalism, which shows how the worker degenerates to the level of a beast at the same time that the machine becomes ever more complex, Stiegler’s [38, 39] history of modern technology paints a nightmarish picture of the dehumanisation and objectification of men and women who seem to have no place in the new world. In much the same way that Lacan [25] reroutes the Freudian idea of the unconscious through Wiener and Shannon in order to show how humanity is part of an enormous computational system, Stiegler [7, 37, 39] imagines the human lost in a high tech civilization no longer worthy of the name, with the important difference that Stiegler thinks we need to find some way to escape from this situation. Akin to Heidegger, and before him Marx, Stiegler [37, 39] suggests that we can trace the origins of this problem back to modernity and perhaps the moment Nietzsche’s Zarathustra first ventured out of his cave. In *Thus Spoke Zarathustra* [44] Nietzsche imagines that his prophet’s insane vision of the end of the God would liberate the ubermensch to make their own law, but Heidegger’s [42] criticism of Nietzsche was that Godlessness had simply produced a technological universe set on blind becoming and little else. In this respect, modernity becomes about the will that wills to will and nothing more. There is nothing beyond what Schopenhauer and Nietzsche wrote about in terms of the will and Freud would later call drive. Although Nietzsche [45] thought about the endless will through the idea of the eternal return, and making the most of whatever comes one’s way, we know that both Freud [20] and later Heidegger [42] saw the terminal downside of Nietzsche’s vicious circle in the infernal mechanisation of the death drive/technological will.

Extending this Freudian/Heideggerian critique, Stiegler sets out to trace the development of the drive-based society up to the present. In his works on *Disbelief and Discredit* [46–48] he draws upon Max Weber’s [49] Nietzschean theory of the spirit of capitalism which, we might recall, explains how the American Calvinists kick-started capitalism in the name of the glory of God. However, we also remember that Weber shows that this was a fatal strategy, at least as far as the longevity of God was concerned. According to Weber, God was over the moment this happened. What this means is that the value rationality required to make money in order to ease the salvation anxiety about whether one was saved or damned in God’s great plan eventually started to undermine belief in God Himself resulting in the emergence of a form of capitalism based upon instrumental rationality. In other words, the capitalists had no more need for God. In this new system the point of making money was simply to make even more money and we can start to see the outline of the drive-based economy. In the first instance, the Protestant work ethic stood in for God, but Stiegler [48] explains that eventually this relic of the religious system was replaced by consumerism, where one works in order to make money in order to consume in order to live in the late capitalist utopia of the fully-satisfied consumer, star, or in contemporary society, the celebrity who is exactly like the rest of us, but somehow better. Thus, Stiegler [48] updates his history of the estrangement of humanity from modern technology by moving through Marx, Nietzsche, Heidegger and Weber until finally he reaches Adorno and Horkheimer’s critique of the culture industry, which becomes about the way that the meaningful structures of culture that would have once held people in webs of significance collapse towards a mechanical production line of meaningless things and the desire for a better future short-circuits towards drive where we want everything now.

In the mid-twentieth century Adorno and Horkheimer [5] were able to show how the culture industry functioned like a kind of capitalist religion, with the consumer in cast in the role of a modern day Tantalus endlessly looking for the commodity that they believe would make them complete, but the problem with this system is that it eventually suffered under pressure of the massive expansion of the economy. At this point Stiegler [50] shows how late capitalism, which is late because it is fatally set on its own self-destruction, starts to consume itself by (a) speeding up innovation, development, and production in the name of increasing profits, (b) relaxing its moral parameters so that more or less anything the consumer wants is for sale, and (c) making the commodity available to pretty much anybody with access to easy credit. Under these conditions, Freud’s Oedipal law, where one is prohibited from ever reaching what one really wants, starts to break down before the increasing speed of the cycle of production, consumption, production, until the consumer enters the space of drive, where the endless line of commodities no longer convince or capture the imagination and they start to see what they really want. Here, Stiegler [50] shows that the more readily available and the more disposable the commodity becomes, the weaker its ability to command its legions of believers, until eventually nobody really believes any longer. In this sense what Stiegler calls ‘disinhibition’, or the collapse of Oedipal law, sounds the death knell for the theological, consumer utopia and the rise of a kind of post-social or asocial society premised on hyper-consumption, disposability, disbelief, cynicism, and a complete disregard for rules and regulation. In recent works, including *States of Shock* [50], *The Automatic Society* [51], and most recently *The Age of Disruption* [7], Stiegler explains that it is precisely this situation that Adorno and Horkheimer [5] saw coming when they wrote about the dialectic of the enlightenment and the cunning reversal of reason towards barbarism through the circuits of instrumental rationality. Now it is under these circumstances that Stiegler [7, 50, 51] thinks that the human, who evolved through tool use and the creation of machines, starts to regress towards a new pre-/post-human state of instinctual/automatic behaviour. However, this new state of second nature no longer resembles the kind of Hobbesian savagery Freud might have imagined, because in the new (un)world instinct is entirely mediated by cybernetic systems that exert control over the pre-/post-human lost in a state of disorientation on the level of behaviour. This is, of course, precisely the kind of system it is possible to identify in Lacan’s [27] re-reading of the Freudian [20] concept of drive which shifts from being a model for understanding the potential degeneration of humanity in Freud’s study of the other side of the pleasure principle to a prophecy about humanity’s technological future in Lacan’s seminar.

Apart from normalising drive, we remember that Lacan’s [25] other major move in his seminar on *Beyond the Pleasure Principle* was to reduce complex language to the level of binary code, with the result that he ended up encrypting death itself in a kind of endless switching machine where the subject is either (a) in full possession of the symbolic representation of the lost object that they take for the thing itself or (b) in a state of despair following the realisation that what they thought was the thing itself is nothing more than a symbolic representation of it leading to (c) the renewal of the search for the thing itself which invariably leads back to (a) and so on ad nauseam. In Stiegler’s [7] work this reduction of the subject to the level of binary code (Boolean man) is reflective of the late capitalist obsession with number and economic understandings of the world through the lens of more or less calculations that eventually result in the emergence of a new computational legal system Rouvroy and Berns [52] write about in terms of algorithmic governmentality. The essential problem of this situation for Stiegler [7] is that it transforms the human into a machine capable of little more than repetition, counting, and basic logic and reduces the thickness of civilized understanding of and engagement with the world to technological hyper-control through data collection and behaviour management. Under the regime of computational hyper-control, there is no deep understanding or moral sense of the need to follow rules. Indeed, Stiegler [7] says that this becomes impossible in the universe of number because endless counting sees memory break down and the basic structure of temporality that enables humans to make sense of their world collapse. In other words, the now robotised subject has no sense of the past to inform their understandings of the present to help them imagine possible futures in advance of their existing situation and instead finds themselves in a situation where there is nothing beyond programmatic repetition and reaction to behavioural switches. We are now in the realm of late capitalist cybernetic law where number is everything and culture means nothing.

As Stiegler [7] explains through reference to Winnicott, culture should enable the subject to transition between existing and future states, but this form of development that enables children to become adults has now been boiled down to blind becoming where the differences between ‘then’, ‘now’, and ‘what is to come’ is expressed numerically or through ideas of cost, benefit, advantage, and profit. Throughout his work Stiegler [50, 51, 53, 54] writes about this situation through the concepts of systemic stupidity, symbolic misery, or, in his recent *The Age of Disruption* [7], ‘the epoch of the absence of epoch’, which suggests that the current historical period is a period paradoxically devoid of all sense of periodisation, history, purpose, or common horizon. Although Hegel, Kojeve, and Fukuyama each imagined the end of history in more or less utopian terms, Stiegler’s [7] end is entirely dystopian in its paradoxical endlessness, which reveals the nightmarish consequences of Lacan’s [25] ‘dehumanisation of death’, at least insofar as this condition is reflected in the new computational utopia/dystopia. At this point in *The Age of Disruption* [7] Stiegler returns to Heidegger [15] and the concept of ‘being towards death’ to explain the impact of the dehumanisation of death and the related emergence of the idea of the endless cybernetic feedback loop upon human life, motivation, and possible futures. He concludes that the effect of the transformation of the end into the endless feedback loop is the profound collapse of the temporal symbolic structures that enabled the construction of self, other, and collective in what he calls the murderous disarticulation of ‘I’ and ‘We’. Caught in this state of collective disindividualisation that results in the subject falling into a kind of schizophrenic crisis, where it starts to doubt its own existence, the only way out appears to be through the kind of fatal circuit Lacan [25] outlined in his re-reading of Freud, which is precisely what find when we explore the way Homo Digitalis looks to endlessly insist upon their own existence through the repetitious posting of selfies on Instagram and other online platforms where the only objective is to say to the world ‘Look at me, I exist’.

Regarding this phenomenon, Byung-Chul Han [55] explains that Homo Digitalis is not simply nobody, but instead should be thought about as somebody with a profile desperately vying for attention in order to confirm their existence. Unfortunately, this desperate need to stand out, to be recognised as somebody, is fatally undercut by the state of isolation that marks life inside what Han calls ‘the swarm’. In a similar vein Stiegler [7] writes about subjectivity in a state of Cartesian anxiety about existence reflected in a desperate need for some sense of recognition in a society that reduces everybody to the fate of the number. Reflecting upon the way this condition impacts upon young people in the process of developing their sense of self hardest, Stiegler writes [47] about the phenomenon of negative sublimation where the ruined individual looks to leave their mark on the world through violent, destructive acts. In his view the deep sense of nihilism that leads to the violence of negative sublimation is what really confirms that we have put an end to the future. Quoting fifteen-year-old Florian—who says ‘You really take no account of what happens to us. When I talk to young people of my generation, who are about two or three years older or younger than me, they all say the same: we no longer have the dream to found a family, to have children, or a profession, or ideals, like you did when you were teenagers. That’s all over, because we are sure that we will be the last generation, or one of the last, before the end’ [7, p. 9]—Stiegler concludes that we must find some way to escape the endlessness of drive and rediscover hope in the future. Although neither Han or Stiegler refer to Lacan’s [25] reading of Freud’s *Beyond the Pleasure Principle* [20] or the stories of Edgar Allan Poe in their work, there is something deeply troubling about the desperation of selfie culture, the phenomenon of negative sublimation, and the need to insist on the existence of the ‘I’ in the face of a situation that seems to run counter to any form of recognition, which recalls the horror of Poe’s [32] Valdemar where the main character’s confirmation of a minimal level of existence resides in knowing that he is already dead. Is this the best Homo Digitalis can hope for in a the new (un)world that has been more or less completely colonised by number and what Zuboff [6] calls surveillance capitalism? This is exactly what Stiegler [7] suggests. Indeed, his suggestion is that we must face up to the horror of this situation and understand that the space of the individual, the inter-individual, or the trans-individual that would have once taken the form of durable culture informed by memories of the past and visions of the future, has now been completely colonised by the data economy. In his view ratio is the new logos meaning that we can forget about the Freudian idea of the moral law. There is nothing left but number and endless counting.

In this respect perhaps Lacan [25] was right about the rise of the cybernetic/computational law, but wrong about the normality of this situation, unless we take the next step of connecting normality with madness, which is, essentially, what Stiegler does across a number of books, but particularly *The Age of Disruption* [7], where insanity is the result of the globalisation of the Californian business model committed to high tech innovation, shock, disruption, and the destruction of all forms of consistency. According to Stiegler [7], the most serious of these destructive acts is the one that transforms culture into a computational rats maze, or behavioural utopia where thinking is replaced by reflex and reaction, because this starts to undermine humanity’s ability to understand reality and recognise its key difference from the machine, which resides in its inability to overcome the barrier of death. While this looks very much like a disadvantage from the side of the machine, it is, of course, very precisely this evolutionary advantage what enables humans to think and make sense, which is why the separation of Lacan from Freud over the issue of the death drive has such important implications for the politics of psychoanalysis, in the sense that by taking the side of the machine and dehumanising death Lacan essentially condemns humanity to a state of pre-/post-humanity where instinct, automation, or what Heidegger [15] called busyness (besorgen) rule, leaving no space for the mobility of thought that enables the human to change their mind. This ability to think differently is particularly important today because, as Stiegler [7] shows, it may be that the post-human technological system has reached its limits in the discovery of the anthropocene, which is generally thought to refer the idea of a completely humanised world, but in actual fact should be understood in terms of the emergence of a pre-/post-human planet, since we now know that the complete humanisation of the earth is in the process of rendering the world unliveable for humans who, we must not forget, rely on the biosphere for their survival.

Responding to this situation Stiegler [7] notes that there is nowhere else we can escape to from here, no more development, no more technological fixes, but the neganthropocentric realisation that we are not machines that can somehow live without bodies and a world that keeps us alive. In essence Stiegler’s ‘survival project’ revolves around this insight, this change of mind, this thinking through of technological estrangement, which he hopes has the potential to transform the techno-post-human anthropocene into an organic-pre-human neganthropocene animated by a culture that rejects the hubris of the Prometheans who think that we are somehow divine beings able to live without bodies that wither, decay, and die in favour of the spirit of Epimetheus that recognises our essential fallibility, vulnerability, and limitations, which are fundamentally linked to the law of all organic creatures. This law says that despite everything, we eventually die. Although this change of mind seems impossible, and the reader will probably doubt that we will ever give up on our machines that are progressively destroying the planet and everybody and everything that relies on it for survival, Stiegler [7] thinks that we will eventually be forced to comes to terms with our limitations, and our own mortality, in the inescapable recognition of the signs of the end times that have been piling up since the early 1970s: the realisation of the limits to economic growth, the end of history, the end of the end of history, 9/11, the financial crash, ever increasing inequality, impending ecological disaster, and now the coronavirus. In this respect, Stiegler sees the potential for the emergence of the incalculable from calculation, finds hope in the hopeless, and the possibility of utopian escape from the nightmarish dystopia of computation that appears endless in its ability to keep counting.

Writing about his own depression in *The Age of Disruption* [7], Stiegler finds a model for a pharmacological response to these signs of the apocalypse (a word which we might recall comes from the Greek ‘apocalypsis’ meaning uncovering or revealing), which would see the original life support system of technology that has now completed its transformation into a kind of global death drive, turn back towards a more sustainable and liveable cultural form. In thinking about the possibility of this new more/less human social order, Stiegler explains that we might find reasons for living that are unthinkable in the contemporary nihilistic system, but returns to the key point over and over again that this potential relies on recognising the inescapable necessity of death and indeed, what we face today, the extinction of life on earth. Thus Stiegler’s survival project, which essentially involves the psychoanalytic possibility of humanity changing its mind, of thinking up some other way of living, and escaping the closed loop of the cybernetic system that has turned the transformative potential of death into a kind of dreary endlessness, rests on confronting the truth of the nightmare that one day there will be no more days. The reason this is so important is because Stiegler thinks that facing up to the nightmare that we are sleep walking towards the end of humanity will open up the possibility of dreaming and imagining ways to realise the dream of escape from our current dystopian situation, similar to the way in which Bataille’s [56] first humans raised themselves above the level of the beasts by painting pictures on the walls of the caves at Lascaux.
